# On the Diseases and Injuries of Seamen

**Published:** 1853-12

**Authors:** G. R. B. Horner

**Affiliations:** Surgeon U. S. Navy, Philadelphia


### MEDICAL EXAMINER,

AND

### RECORD OF MEDICAL SCIENCE.

NEW SERIES.—NO. CVIII.—DECEMBER, 1853.

### ORIGINAL COMMUNICATIONS.

### *On the Diseases and Injuries of Seamen.* By G. R. B. Horner, M. D., Surgeon U. S. Navy, Philadelphia.

#### *Diseases of the Respiratory Organs.*

Notwithstanding the strict examination of seamen before their admission on board ship, there are no class of persons who suffer more from the above complaints. This may be ascribed mainly to their great exposure to every vicissitude of weather, their often being wet with rain or sea water, neglecting or not being able to change their clothes, and when chilled not having it in their power to warm themselves by any artificial means. When on watch, too, they are very much habituated to lying down on the decks, and even beneath the sails, reflecting down upon them the strongest currents of air. Likewise, seamen when sleeping below decks, and the air presses down through the hatches and into the ports, are very liable to take cold. This, moreover, affects them from its constant application, and being accompanied always with more or less dampness, either in the external air, or in that of their vessels. These likewise shift like the weather, and so often pass from hot to frigid climates that their crews suffer thereby as they would from exposure to vicissitudes of weather, and when remaining in any fixed place. Owing to these causes seamen are liable to every species of disease which effects the lungs, their air-pasages and investing membranes. From neglect and want of proper medical attendance, many of them are prone to be severely and chronically diseased; organic changes necessarily follow, and cures are more difficult of attainment. Upon the inspection of the diseases which occurred in three men-of-war—two frigates and one ship of the line—of which I was surgeon, I have found that in one of the former 265 cases of catarrh, alone, took place. In the other frigate there were 106 within 18 months, the period I was on board her; and in the Delaware, the ship of the line referred to, which had a crew of 840 persons, 232 cases occurred, although she spent more than a year between the tropics before she went to the Mediterranean. During the entire cruise of two and a half years, 23 of her crew had phthisis pulmonalis, of whom twelve died; eight on the Brazil station, and four after she left. But one of the former died in a merchant vessel before she got clear of the coast, and one of the latter either at our naval hospital, Port Mahon, or else in returning home. Of the crew of the United States, the first frigate referred to, ten had phthisis, of whom eight died, four on board, and four at the above hospital under charge of another surgeon than myself; but in the Savannah, the second frigate, merely three cases of the disease happened, of which only one was fatal, that of a supernumerary who came on board of her in California for treatment. The comparative exemption of her crew from the disease is ascribed to the great care in selecting from it, and sending on shore at Boston, before she sailed, all persons who were believed to be of unsound constitution. The great reduction in the number of her crew on her arrival in the above country, also served to render the number of cases proportionally less, and we must likewise ascribe the small amount of these and other cases of pulmonic diseases to the avoidance of wet decks, keeping the vessel continually of moderate temperature by the use of stoves in her, and the receiving ship, Franklin, to which the crew were transferred while the Savannah was in the dry-dock undergoing repairs. But the cold was so intense, the air so humid, when the wind blew from the north-east and east, that eleven of them were affected with pleurisy, between the 12th

January and the 4th of March, 1849, the period included within the time of her arrival off and departure from Boston. No deaths took place among these cases, and a very small number of other pulmonic ones, except phthisis, in the above and other vessels terminated fatally. Among these cases was the one of pneumonia, in the Brandywine, followed by mortification of the lungs, and another accompanying a violent remittent fever, off the coast of Syria, and in another frigate. From catarrh no death directly happened, but one of the fatal cases of phthisis was traced to it, and one of hypertrophy of the heart in the Delaware which ended in like manner,two months after the patient contracted a catarrh in the form of influenza, which attacked the crew in the first week of September, 1843, while she was at Naples. More than sixty of them were affected at the same time with the complaint to such a degree as to be rendered unfit for duty. Many others were affected moderately and were not taken on the sick list. What was most remarkable respecting the disease, was, its prevalence during warm and fair weather, without any great change of temperature, Fahrenheit's thermometer having been at 76°, the day before the influenza appeared; at 73° on the same day the three first cases were admitted, and only at 71° upon the 10th of September, or three days afterwards. The thermometer fell a fraction on the day the disease appeared, and at the same time the wind blew freshly from off shore, and the Appenines, and raised by its great dryness the hygrometer to a most extraordinary height. As no rain had recently fallen, a whitish impalpable dust was blown to the ship, covered her rigging, and caused the sailors to think it was sulphur ejected from Mouut Vesuvius, although the wind was north-east, and the latter bore almost south-east. They likewise concluded that the dust being sulphur must have occasioned the epidemic.

On board the United States the atmospheric phenomena were different. The air had been damp and chilly, the wind was variable; the thermometer was at the freezing point the day we entered the gulf of Smyrna, and snow covered the adjacent mountains. But on the 26th of January, 1837, the day before she left that port, and three weeks from the former period, the thermometer was at sixty-one degrees. The number and violence of these cases in this vessel corresponded with the extremes in temperature. One hundred and twenty-six of the men were affected with catarrh in such a severe form as to have been taken on the sick list between the seventeenth of January, when the complaint began to assume an epidemic form, and the 12th of February, when it ceased to be so, and the vessel arrived at Port Mahon.

On the 27th of January ninety men, including officers, were on the list, and almost the whole number had the complaint, some moderately, others violently, and accompanied with high fever, acute pains in the head, chest and limbs, and delirium. Among the latter was an officer liable to hereditary insanity, and who has been a maniac ever since. His mind had been previously wavering between sanity and insanity, and lost its balance when catarrhal fever attacked him. In these cases, as well as those of the same kind in the Delaware, venesection was employed: leeches were applied to the temples, cups and blisters to the chest, when it was much affected; pectorals, anodynes and diaphoretics were prescribed; but in a vast majority of patients I effected a cure in a few days, often in a single one, by first giving an ounce of epsom salts and one or two grains of tartar-emetic in ℥viij of water at several doses; then by administering a solution of the latter medicine in the dose of an eighth of a grain in simple water, but generally, as being most palatable and soothing to the throat, when flavored with sugar, gum arabic and extract of liquorice. This pectoral was given every hour, or at intervals of three or four hours, and was aided by some hot herb tea, as that of catnip, marsh-mallow and horehound. Balm and eupatorium are also freely used by me in such cases, and for producing copious perspiration the latter is unrivalled by any of the other herbs mentioned. To promote the diaphoretic effects of all of them, the feet were well bathed in hot water; and as this also produced a revulsive effect from the brain, it composed this and caused the sick to sleep soundly. The bath, moreover, helped to relieve coryza, which was often very profuse, from the inflammation of the mucous membrane of the nostrils being very intense; but in these cases, the direct application of steam or hot water to them several times a day was most effectual, from its inducing a profuse secretion of mucus and disgorging the capillary vessels. A few grains of super-acetate of lead or sulphate of zinc render the hot water still more effectual. The steam of vinegar or brandy is likewise beneficial, but I prefer that of water. This, moreover, I have found best to relieve otitis, which sometimes attends catarrh, and for its cure may be applied by means of a hot brick wrapped in a damp towel.

Several cases of pleurisy and one of pneumonia occurred in the United States at the same time with the influenza, and were cured by copious venesection, cups and blisters to the thorax, and other remedies enumerated. In the treatment of the last named affection, the spiritus mindereri and neutral mixture with tartar emetic dissolved in them, and the nitrous powders containing this medicine, in the quantity of an eighth of a grain, with one grain of calomel in each powder, produce a happy effect. The three first named articles promote sweating and expectoration, open the bowels and reduce the pulse, while the last named decreases the plastic property of the blood and causes the absorption of any effusion which may have occurred in the lungs, or within and upon their pleural coat and that of the ribs. By observing the above practice with some variations, none of the above cases of pleurisy and pneumonia ended fatally, and no others, except two private ones to which I was called in the last stage, and which had been unattended by any physician. One was that of a marble mason, who persisted during a rainy, chilly day in polishing one of the columns to the portico of the Naval Asylum A violent inflammation of the lungs, bladder, and almost every tissue of his body supervened ; and having been allowed to rage unchecked, every affected part became so injected with red blood as to appear to have been done by artificial means. The mucous coat of the bladder was the most peculiar, from being of a scarlet hue, covered with dark red spots.

The other case was that of a citizen of the island of Milo, south of Greece, and to whom I was called during a transitory visit to the town, in April, 1833, while surgeon to the John Adams. Although the island contained some thousands of inhabitants, there was not then a single physician among them. The patient had therefore received no proper medical attention, was in the last struggle for existence, and gasping loudly for breath in a low bed and at the back of a dark room. A crowd of women, wrapped in white muslin mantles and dresses, stood around the bed, and holding flaming torches in their hands, were mournfully looking on at the dying man. He laid, apparently unconscious of their presence, upon his back, making imperfect inspirations and expirations; his face was sunken, pallid, cadaverous, his pulse was rapid and bounding, but sinking. There was not the least hope of his recovery, yet I prescribed, through an interpreter, what seemed calculated to comfort him, and had to hurry away before the awfully tragic scene had terminated, lest darkness should overtake me before the landing place and ship were reached. Her distance from the shore, and this from the town, prevented me from making any attempt at a post-mortem examination, and had it been convenient to make one, the friends of the deceased would probably have refused it. From the symptoms, too, the morbid changes, I judge, would have been similar to those just described, from this man having had the disease also unchecked. The changes found in the dissection of the seaman, mentioned as having died of hypertrophy of the heart succeeding catarrh, were the following: The whole organ was much enlarged, and especially its left ventricles, the parietes were thickened, the auricular ventricular openings expanded, the chordæ tendinæ and columnæ carnæ elongated and thickened, the aorta was expanded and gritty, the pericardium contained several ounces of serum, and the left ventricle was filled with dark blood. The right lung adhered to the ribs; its bronchia were expanded; it contained some tubercles and purulent matter, and within the pleura were about two pounds of serum. The liver was indurated, of a greyish red color like porphyry, and much engorged. The gall bladder was small and filled with dark bile; the pancreas indurated and enlarged; the mucous coat of the stomach congested and somewhat softened.

From these morbid alterations, his age, which was fifty, his habits as a sailor, and his not having complained before attacked with catarrh, of any cardiac, or other disorder, to my knowledge, the following conclusions were drawn: that by long continued indulgence in ardent spirits, the liver, pancreas and stomach had been affected, and the two first indurated insensibly; that the circulation of the blood having been partially interrupted, the heart then commenced to enlarge, and the influenza afterwards having seized him, that organ, the right lung and pleura became actively inflamed. This was very clearly indicated by the great fulness, frequency and strength of his pulse, great dyspnœa, pain in the chest and other symptoms, which caused me to bleed him copiously twice within the first three days after he was taken on the sick list, to bleed him moderately once towards the close of his life, and to administer the tincture of digitalis, squills, antimonials, tr. assafœtida, extract of stramomium, and other anodynes, besides making local applications over the diseased parts.

In the treatment of phthisis, of the tubercular or other kinds, the above remedies with a variety of others were employed, but the result as stated has already shewn that they often failed in my hands as they have done in those of many more physicians. Relief was certainly obtained by using these remedies, the patients were at least made more comfortable, but in all climates consumption had its full quota of victims, in spite of cod liver oil and every other specific recommended. But in some cases I was gratified by success. Two of them were, that of a marine who had been completely prostrated by phthisis and its accompanying chronic diarrhœa, and the other that of a seaman just returned from a cruise in the East Indies ; but his case was not one of tubercular phthisis, and was thought to have been induced chiefly by an adhesion of the upper convex surface of the liver to the diaphragm and inflammation extending from it into the right lung. An abscess formed in this, a large quantity of thick whitish pus was discharged day after day, and finally became so tinged with green, as to permit no doubt that bile was being effused from the liver through an ulcerated passage extending through the diaphragm into an adhering portion of the lung. This patient, John Ward, was and is now, a pensioner in the Naval Asylum, of which I was surgeon when he was treated. He was cured chiefly by means of tincture of digitalis, gum ammoniac and other expectorants, blisters to the chest, tartar emetic ointment as a dressing, and pills of blue mass. The other case was cured, at least sufficiently for him to resume his duties, by the internal use of a solution of sulphate of zinc and sulphate of morphine, in the proportion of one grain of the former to $\frac{1}{8}$ of the latter 3 or 4 times a day in a half ounce of water, and by injecting the same compound into the rectum to check the diarrhœa.

While I was at the Naval Asylum, another uncommon instance of phthisis occurred in the person of Wm. Williams, a one legged pensioner, of intemperate habits. He was under treatment for six months, had taken various pectorals, composed of tinc. of tolu, gum ammoniac, acetate and sulphate of morphine, had worn a tartar emetic plaster, and for a long period had his wasting frame supported by bitter infusions and quinine mixture. In the mean while he expectorated a large quantity of pus, and discharged almost as much from an abscess over the anterior face of the right ribs. Upon death having occurred, an autopsia was made to ascertain what connection existed between the internal and external abscess, and to discover what other morbid changes existed. Four of the true ribs, the third, fourth, fifth, sixth and seventh, were found to have formed the base of the external abscess, and beneath the third and fourth ones was the abscess in the lung. No direct communication was discovered between the two abscesses, but it was thought that the trace of it had been removed by the knife and saw used in dissection. The cartilages were ossified, and one was separated from its rib by caries. This was plainly owing to the pulmonic abscess behind it having had its contents brought into contact with the bone and cartilage. The external abscess having discharged its fluid contents through the fistulous orifice which had so long existed, contained only some cheesy matter. The right lung had a small quantity of water effused in it, and the left lung had much contracted in size, was filled with tubercles of a blackish grey color, adhered closely to the ribs and cartilages, so that the pleura costalis and pulmonalis were merged into one and undistinguishable, and the lung in its antero-posterior diameter was merely about one inch and a half in diameter. The liver was very small and blanched at its lower edge, but the right lobe was elongated downwards into a flap three inches long, and its structure otherwise normal.

In conclusion I will state concerning this patient, that he had an attack of hæmoptysis a year before he was treated for phthisis, and was nine days under my care for the former.

Six other patients at the Asylum besides those spoken of had phthisis during my last term of service there, and one died of it there before it was opened for the reception of pensioners, and while I was assistant to our late distinguished chief of the bureau of medicine, Dr. Thomas Harris. The latter patient was a seaman who, while dying, nobly offered, without any request or persuasion, to let me "do with his body after death as was thought fit." Accordingly it was examined. His lungs were found filled with tubercles, but the right one, as I have seen in most dissections, was worst diseased, containing several vomicæ, and being totally disorganized. Of the six other cases three ended in death, and one within twelve days after it was treated. This case was an obscure one. The patient was 65 years old; came on the list, and was treated for a cough and diarrhœa of great obstinacy, which seemed the chief cause of death. He was much prostrated, and in conjunction with an expectorant and opiates taken by mouth and used as enemata had to be prescribed, pills of sulphate of quinine, and an infusion of pulv. cinchonæ, colombo, ginger and orange peel. For cough, and shortness of breath of an asthmatic kind, he was also given a tartar emetic plaster to be worn over the chest. The infusion having appeared to purge, was combined with laudanum, and xx. drops were taken in each dose. Opiate enemata were prescribed pro re nata, the diarrhœa having become worse. It was impossible by these or other medicines to sustain him; the stools became watery, his excretions of all kinds offensive, and he expectorated some greenish pus ten days before his decease. Upon examining him, both lungs were found adhering to the ribs, filled with tubercles, collapsed and shrivelled. The right lung, however, as expected, was worst, for it contained many vomicæ of small size, and adhered to the ribs by strong tendinous bands. The small intestines and stomach were inflamed chronically, and the latter had its mucous coat atrophied, of a dark red color, and deprived of its cryptæ and folds. The liver was blanched and indurated, and had its acini indistinct. But the most unnatural phenomena were the thickening and softening of the parietes of the heart, and the perfect adhesion of this to the pericardium, no interstice having been left between them. No instance of this kind had ever before fallen under my notice, nor has another been since seen.

Another pensioner, named Wilhelm, a foreigner, had long been gate keeper at the Asylum, and was remarkable for his florid complexion, slender and agile form. He came on the list with a slimy diarrhœa and inflamed eyes. His tongue was thickly furred, his abdomen sore to the touch, and he had had a cough for some time ; but little notice was taken of it until he had been on the list for three weeks and expectorated a quantity of glairy, greenish mucus. The stethoscope was then applied, and detected a mucous rattle. His bowels up to that time had attracted chief attention, and had been restricted by first giving a dose of pulv. rad. rhei, magnesia and laudanum, and afterwards several sorts of pills, besides some grains of kino and tinc. cinchona compos. as he had been a free drinker. The pills were of ipecac. and opium at first, then of these articles and blue mass, and last of the former, calomel and essence of peppermint. Two days after his admission he complained of being very weak, and was directed two doses a day of a quinine mixture and a pill at night. He likewise was given an infusion of cinchona, colombo and ginger, with tincture of opium, a brown mixture for his cough, some opiate enemata, pills of quinine, opium, and essence of peppermint; had a Burgundy plaster put on his chest, and a flannel bandage around his abdomen. Nothing availed ; he died twenty-five days after taken in charge.

*Autopsia.*—Two ounces of serum were in the pericardium, the lungs adhered in every part to the ribs ; when cut into resembled greyish granite, were filled with tubercles beginning to suppurate, but the right lung was most diseased. The stomach was distended with flatus, its mucous coat was deficient, being about the thickness of brown paper, was softened, and easily scraped off with a knife handle ; the folds and villi were almost invisible, and slight inflammation existed in the cardiac orifice. Three ulcers were seen in the jejunum within a foot of one another, and completely perforating its coats ; the peritoneal coat of all the small intestines was inflamed, slightly in most parts, but greatly in some from the effusion of fæces into the cavity of the peritoneum. The liver was enlarged, indurated, nearly white, and deprived of its acini ; the spleen was indurated, but weighed only about three ounces, was merely two and a half inches long, one thick, and one and a half broad, and contained no grumous blood, The bladder was contracted and blanched, its coats were thickened, the left kidney was enlarged, of a light red color, and nearly as hard as dried beef, and the right kidney, though of natural size, was of the same consistence.

Such were the effects of disease on this poor old sailor, aggravated by a long-continued abuse of ardent spirits—the greatest of all curses to his fraternity, and destroying more of them than become victims to the tempests or the billows of the ocean.

